# The effect of a brief intervention video on gambling advertising resistance: Results of a randomized, on‐line experimental study

**DOI:** 10.1111/add.16732

**Published:** 2025-01-14

**Authors:** Jamie Torrance, Alex M. T. Russell, Conor Heath, Philip Newall

**Affiliations:** ^1^ School of Psychology Swansea University Swansea UK; ^2^ School of Psychology University of Chester Chester UK; ^3^ School of Health, Medical and Applied Sciences, Experimental Gambling Research Laboratory CQ University Bundaberg Australia; ^4^ Faculty of Computing, Engineering and the Built Environment Birmingham City University Birmingham UK; ^5^ School of Psychological Science University of Bristol Bristol UK

**Keywords:** counter‐advertising, gambling advertising, gambling marketing, intervention, persuasion, scepticism

## Abstract

**Background and aims:**

Gambling advertising is nowadays prevalent in multiple jurisdictions and can take multiple forms, such as TV adverts and social media promotions. However, few independently designed interventions for gambling advertising have been empirically tested. We aimed to measure the effectiveness of an inoculative intervention video for gambling advertising, which was developed based on previous interventions for alcohol and tobacco, and which used input from academics and experts by experience.

**Design:**

Between‐participants randomised online experiment.

**Setting:**

UK.

**Participants:**

UK‐based gamblers aged 18–29 years (*n* = 1200) were recruited via Prolific.

**Intervention:**

Participants either saw a novel inoculative intervention video (7.2 mins) aimed at increasing resistance against gambling advertising strategies (*n* = 595) or a neutral control video (7.2 mins) on healthy eating (*n* = 605).

**Measurements:**

Participants completed pre‐ and post‐test measures of gambling advertising scepticism and persuasion knowledge immediately before and after video exposure. They also answered the Problem Gambling Severity Index (PGSI) and reported their past‐month engagement with gambling promotional offers. A random subset of participants (*n* = 797) recompleted these measures at one‐month follow‐up.

**Findings:**

The intervention group's post‐test scores were statistically significantly higher than control for scepticism [estimated marginal means (EMM) = 40.32 vs. EMM = 34.72; *P* < 0.001, 95% confidence interval (CI) = 4.90–6.29, ηp2 = 0.17] and persuasion knowledge (EMM = 20.77 vs. EMM = 16.71; *P* < 0.001, 95%CI = 3.61–4.50, ηp2 = 0.21). One‐month follow‐up scores also remained statistically significantly higher in the intervention group compared with control for both scepticism (EMM = 38.26 vs. EMM = 34.73; *P* < 0.001, 95%CI = 2.70–4.36, ηp2 = 0.08) and persuasion knowledge (EMM = 18.63 vs. EMM = 17.21; *P* < 0.001, 95%CI = 0.88–1.95, ηp2 = 0.03). Within the intervention group, 21% of participants had stopped engaging with gambling promotional offers at one‐month follow‐up, reflective of a statistically significant reduction compared with control (EMM = 0.48 vs. EMM = 0.87; *P* < 0.001, 95%CI = −0.53 to −0.26, ηp2 = 0.04). Overall, the control group showed no statistically significant changes in any of their scores throughout the study period.

**Conclusions:**

An inoculative intervention video appears to increase young gamblers' resistance to gambling advertising and reduce their self‐reported engagement with promotional offers.

## INTRODUCTION

Counter‐advertising interventions can develop consumers' resistance against the persuasive marketing strategies of the alcohol [1, 2] and tobacco industries [3, 4, 5], among other harmful industries [6]. Counter‐advertising interventions are often grounded in ‘inoculation theory’, which posits that resistance to commercial manipulation is developed via exposure to weakened forms of this manipulation [7, 8]. For example, the United States (US) ‘Truth’ campaign included examples of tobacco advertising to demonstrate the tobacco industry targeting of younger audiences [5], and fostered a 22% decrease in youth smoking rates between 1999 and 2002 [9]. In a more recent study, an inoculative counter‐advertisement focusing on alcohol sponsorship in sports increased knowledge of alcohol harms and led to lower drinking intentions among Australian rugby league fans [1]. However, although recent research has highlighted the similarly persuasive elements within gambling advertising [10, 11, 12], we know of no previous research adapting inoculation theory‐based interventions to gambling.

Owing to the relaxation of regulations over the past two decades, gambling advertising is now prevalent in various jurisdictions, including the United States [13], Ireland [14], Spain [15], Nigeria [16], Australia [17] and the United Kingdom (UK) [18]. Gambling advertising occurs in various forms, including standalone commercials on television and the internet, embedded promotions in sports, direct texts/emails to consumers and promotional social media posts by operators and affiliates [19, 20]. This has led consumers to report their environments being ‘saturated’ with ‘unethical’ and ‘deceptive’ gambling advertisements [18, 21]. Gambling advertising's persuasive strategies include the positive framing of gambling, where gamblers are falsely shown as predominantly winning [19]. These advertisements are often strategically constructed to persuade specific demographic groups. For instance, sports betting advertisements predominantly feature male characters and incorporate notions of team loyalty and humour [22], whereas bingo advertising primarily involves female characters while emphasizing a fun and communal atmosphere [23]. Another persuasive advertising strategy involves a high frequency of gambling logos in professional sports through shirt sponsorship and signage around the pitch [24, 25, 26], thereby piggybacking on fans' strong emotional bonds toward their teams [27].

Another longstanding gambling advertising strategy involves the dissemination of promotional offers (otherwise known as financial inducements/incentives). Previous research has identified at least 15 different types of promotional offers within gambling advertisements such as free bets, sign‐up bonuses and refund/stake‐back offers [28]. The economic value of such promotions is often obscured by esoteric language and strict conditions that are difficult to interpret [29]. Despite this lack of clarity, promotional offers appear to be particularly persuasive in facilitating more impulsive and riskier bets among those exposed to them [30, 31, 32, 33]. Last, affiliate marketing is a common gambling advertising strategy whereby third‐parties receive a commission from gambling operators to promote particular bets or offers via their own social media channels [34]. It is common for affiliates to pose as ‘experts’ or ‘tipsters’ who promote bets that are seemingly low‐risk [35]. However, given that affiliates often receive a revenue share from unsuccessful bets, and that affiliate‐promoted bets have a low chance of success [36], the sincerity and transparency of affiliate marketing in a gambling context has been called into question [34].

The promotion of addictive or harmful products has often been subject to various regulatory interventions whereby harm‐reductive messages are to be displayed within advertisements [37, 38, 39]. In the United Kingdom, gambling advertisements include ‘safer gambling’ messages, but industry‐developed slogans like ‘When the FUN stops, stop’ and ‘Take time to think’ have been criticized for their lack of protective effect on gambling behaviour [40, 41], and for placing the onus of responsibility on the consumer [42]. The UK's Department for Culture, Media and Sport (DCMS) has announced that independently developed safer gambling messages will replace the industry‐developed slogans by mid‐2024 [43]. Existing independently developed messages are also typically aimed at the general harms associated with gambling, rather than the harms associated with gambling advertising in itself [10].

There is a consequent need for independently developed counter‐advertising strategies for gambling advertising, which can complement the forthcoming short‐form messages. Longer‐form interventions such as videos could well benefit from content that promotes scepticism toward the credibility of the advertising source and increases awareness of the persuasive strategies used [44]. Young adults are also a target demographic for gambling advertising [19], because the risk of experiencing gambling‐related harm is high during the initial years of access to legal gambling opportunities [45].

The current study, therefore, aims to experimentally test the efficacy of a brief‐intervention video among young adult gamblers to foster scepticism, persuasion knowledge and resistance in relation to gambling advertising strategies. We aimed to test the following preregistered hypotheses: (1) there will be an increase in scepticism and persuasion knowledge scores from pre‐ to post‐test for the intervention group, with these changes being larger and statistically significant compared to the control group across all Problem Gambling Severity Index (PGSI) groups. (2) At 1 month follow‐up, the intervention group will maintain or increase their levels of scepticism and persuasion knowledge without significant decrease from pre‐ or post‐test and will show significantly lower or not significantly different engagement with promotional offers compared to measurements at post‐test.

## METHODS

### Design

A between‐participants, randomised on‐line experiment was conducted, comprising two conditions (intervention video and neutral control video) across three time points (pre‐test, post‐test and 1‐month follow‐up). The preregistration can be found here: (https://osf.io/gk3by), with the data, materials, additional PGSI analyses and intervention video available from https://osf.io/jb43f/. Ethical approval was obtained from the University of Bristol (17525).

### Participants and recruitment

**FIGURE 1:**
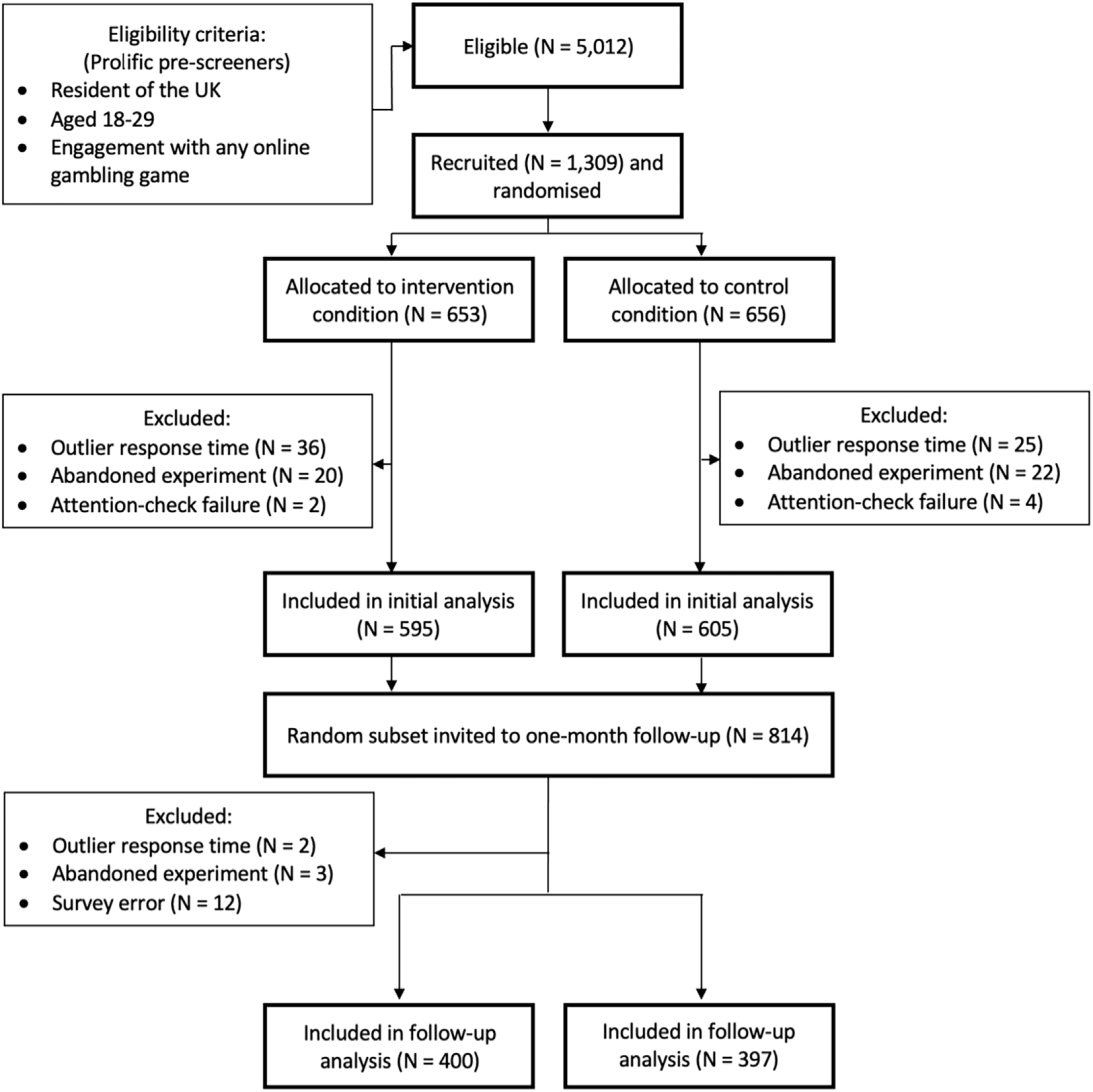
Consolidated Standards of Reporting Trials (CONSORT) flowchart.

A Consolidated Standards of Reporting Trials (CONSORT) flowchart is presented in Figure 1. Participants were recruited via the on‐line crowdsourcing platform Prolific. Participants were compensated £3 for the initial study (mean duration = 14.5 minutes, £12.36 per‐hour pro‐rata) and £1.23 for the 1‐month follow‐up study (mean duration = 2.9 minutes, £25.74 per hour pro‐rata). Only Prolific users who resided in the United Kingdom, had previously indicated on Prolific that they engage with some form of on‐line gambling, and were 18 to 24 years of age were eligible. In a deviation from the preregistration, the maximum age of participants was increased to 29 years on the last day of data collection as few additional participants were being recruited (699 participants were age 25–29 in the final sample). According to the World Health Organisation [46], the 18 to 29 age bracket represents ‘young adults’ and has been used in previous gambling studies aiming to gather data from this demographic [18, 47, 48]. A power analysis indicated a required sample size of 816 participants for comparing two groups (power: 0.95, α: 0.05, effect size: f = 0.1). To account for attrition and in consideration of similar studies [1, 49], we increased the target to 1200 participants, balancing statistical power with the project budget. The initial experiment successfully achieved this preregistered target sample size. Across both studies, outlier responses (*n* = 63) that were three SDs below or above the mean response time were excluded before analysis [50].

The demographic characteristics of the sample were collected via Prolific and are presented in Table 1. Participants' self‐reported engagement with on‐line and in‐person gambling is also presented in Table 1. Participants had a median score of 2 (skewness = 2.1) on the PGSI, which is a standardised tool used to measure at‐risk gambling behaviours in community samples [51]. The Cronbach's α of the PGSI was considered excellent (α = 0.90). Overall, 419 participants (34.9%) were categorised as no‐risk, 310 (25.8%) as low‐risk, 287 (23.9%) as moderate‐risk and 184 (15.3%) as high‐risk gamblers. 1

**TABLE 1:** Demographic characteristics and past‐year gambling engagement split by condition at baseline.

Attribute	No. (%)		
	Intervention (*n* = 595)	Control (*n* = 605)	Total (*n* = 1200)
Age: mean (SD)	25 (2.7)	25 (2.7)	25 (2.7)
Sex^a^			
Male	315 (52.9)	302 (49.9)	617 (51.4)
Female	276 (46.4)	302 (49.9)	578 (48.2)
Prefer not to say	4 (0.7)	1 (0.2)	5. (0.4)
Jurisdiction			
UK	595 (100)	605 (100)	1200 (100)
Ethnicity			
White	443 (74.5)	470 (77.7)	913 (76.1)
Asian	61 (10.3)	47 (7.8)	108 (9)
Black	51 (8.6)	49 (8.1)	100 (8.3)
Mixed	35 (5.9)	32 (5.3)	67 (5.6)
Other	5 (0.8)	7 (1.2)	12 (1)
Employment status			
Full‐time	306 (51.4)	322 (53.2)	628 (52.3)
Part‐time	84 (14.1)	103 (17)	187 (15.6)
Due to start in next month	13 (2.2)	9 (1.5)	22 (1.8)
Not in paid work (e.g. home keeper or retired)	31 (5.2)	23 (3.8)	54 (4.5)
Unemployed or job seeking	59 (9.9)	68 (11.2)	127 (10.6)
Other	27 (4.5)	19 (3.1)	46 (3.8)
Data expired or preferred not to say	75 (12.6)	61 (10.1)	136 (11.3)
Past‐year gambling engagement^b^			
On‐line:			
Sports betting	331 (55.6)	375 (62)	706 (58.8)
Slots	229 (38.5)	239 (39.5)	468 (39)
Lottery	276 (46.4)	275 (45.5)	551 (45.9)
Poker	75 (12.6)	90 (14.9)	165 (13.8)
Blackjack	91 (15.3)	117 (19.3)	208 (17.3)
Roulette	114 (19.2)	133 (22)	247 (20.6)
Scratch cards	176 (29.6)	198 (32.7)	374 (31.2)
Other	70 (11.8)	70 (11.6)	140 (11.7)
In‐person/venue:			
Sports betting	55 (9.2)	51 (8.4)	106 (8.8)
Slots	82 (13.8)	77 (12.7)	159 (13.3)
Lottery	124 (20.8)	147 (24.3)	271 (22.6)
Poker	48 (8.1)	46 (7.6)	94 (7.8)
Blackjack	38 (6.4)	50 (8.3)	88 (7.3)
Roulette	42 (7.1)	47 (7.8)	89 (7.4)
Scratch cards	227 (38.2)	255 (42.1)	482 (40.2)
Other	32 (5.4)	36 (6)	68 (5.7)

Abbreviation: UK, United Kingdom.

^a^ Prolific does not provide non‐binary options.

^b^ Participants could choose more than one answer.

### Measures

#### Gambling advertising scepticism

Gambling advertising scepticism was measured via an adapted version of the 9‐item SKEP [52], which has been used to measure consumer scepticism across medication advertising [53], environmental or ‘green’ claims by marketers [54] and political advertising campaigns [55]. The items were adjusted to address ‘gambling advertising’ rather than ‘advertising’ in general and included statements such as: ‘We can depend on getting the truth in most gambling advertisements’. Responses were measured on a 5‐point Likert scale of agreement (strongly agree‐strongly disagree) with total scores ranging from 9 to 45. Higher scores were indicative of higher scepticism. The Cronbach's α of this measure was good (α = 0.86).

#### Knowledge of persuasive gambling advertising tactics

There is no universal measure of persuasion knowledge, and previous studies have, therefore, used a range of self‐report questionnaires [56]. Persuasion knowledge was operationally defined here as self‐confidence in interpreting strategies commonly used in gambling advertising [57]. We, therefore, provided participants with plain‐language definitions of five gambling advertising persuasion strategies [(1) the positive framing of gambling; (2) targeted advertising; (3) promotion in sports; (4) promotional offers; and (5) affiliate marketing] in line with Jung and Heo [58]. Using a 5‐point Likert scale, participants rated their level of knowledge (none at all–a lot) for each of the five persuasive advertising strategies. Total scores ranged from 5 to 25 with higher scores being indicative of higher gambling advertising persuasion knowledge. The Cronbach's α of this measure was considered excellent (α = 0.91).

#### Past‐month engagement with gambling‐related promotional offers

Past‐month engagement with gambling‐related promotional offers was measured via the question: ‘In the previous month, how often have you used offers provided by gambling advertising? This includes sign‐up/deposit bonuses and “free” bets’. Example screenshots of promotional offers were displayed alongside this question, and participants responded via a 5‐point frequency Likert scale (never‐always).

### Intervention video

**FIGURE 2:**
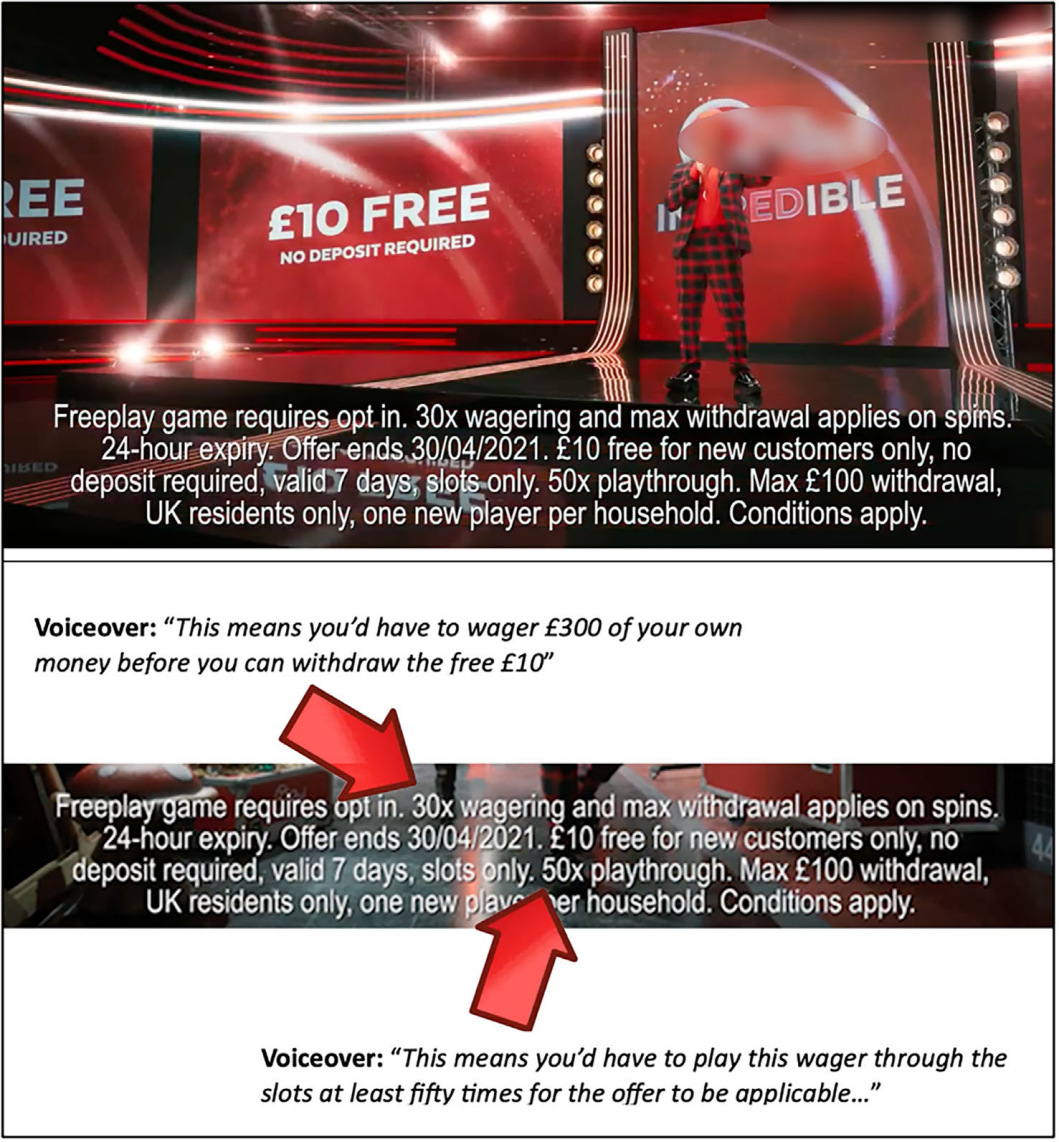
Screenshot (with text detailing the audio voiceover) of the intervention video during the inoculative segment on promotional offers.

A prototype intervention video was developed based on two previous reviews [19, 59], and improved based on feedback from focus groups involving gambling academics and gambling experts‐by‐experience. A thematic analysis of this feedback is outlined in more detail elsewhere [60]. The final intervention video was 7.2 minutes in length and provides inoculative content on gambling advertising strategies from a consumer‐protection perspective, regarding the positive framing of gambling, targeted advertising, promotion in sports, promotional offers and affiliate marketing. Following a brief introduction, the voiceover outlined each tactic, offered a definition, emphasized potential risks, provided example counterarguments to these risks and subsequently presenting logical refutations to these counterarguments. During each inoculative segment, visual examples were provided (Figure 2).

### Procedure

Participants completed the adapted SKEP and persuasion knowledge scales at pre‐test before being randomly allocated to either the intervention video condition or the control video condition. The control video, which matched the length of the intervention video, featured content promoting healthy eating by National Health Service England. The random allocation of participants into either condition was performed using a 1:1 ratio via Qualtrics' built in randomisation software. Random allocation worked as intended given that no significant differences were observed at baseline between the intervention and control group regarding scepticism (*P* = 0.828), persuasion knowledge (*P* = 0.475) and past‐month engagement with promotional offers (*P* = 0.244). Participants were not explicitly made aware of the randomisation process and condition allocation was not mentioned within the information sheet or debrief. However, because of the inherent nature of the video intervention and its alignment with the pre‐/post‐test measures, it was not feasible to blind all participants to their condition allocation in this study.

To minimize inattention, the videos were programmed to pause after approximately 40 seconds. To proceed, participants were required to correctly enter a unique code stated in the video voiceover into a text box beneath the embedded video player. Incorrect input of this code redirected participants to the end of the experiment. However, no participants had their data excluded from analysis because of incorrect code input. The embedded video players were also programmed to disallow navigating or skipping through the video, and progression to the next stage of the experiment was only possible after the videos had played in their entirety.

Following video exposure, participants completed post‐test measures of the adapted SKEP and persuasion knowledge scales as well as the PGSI. Participants also reported the forms of gambling they engaged with alongside their past‐month engagement with promotional offers. As an additional data quality check before submitting their responses, participants completed a self‐reported carelessness and attention measure. Specifically, this measure included the question ‘in your honest opinion, did you pay attention to the survey and should we use your data?’ with two response choices: ‘yes I was paying attention, use my data’ and ‘no I was not paying attention, discard my data’. At this stage, data for analysis were excluded from participants who failed this measure (*n* = 6). After completing this initial study, participants were then redirected to Prolific to receive payment. A random subset (*n* = 797) of participants took part in the 1‐month follow‐up. In general, all longitudinal designs suffer from participant attrition, and this is equally true on Prolific, where one previous methodological study suggested a rate of 76.7% retention at an interval of 12 months [61]. Therefore, our lower preregistered sample size for the 1‐month follow‐up reflected an anticipated proportion of participant dropout, no matter the time or budgetary resources invested in this part of the project. At 1‐month follow‐up, the subset of participants completed the adapted SKEP and persuasion knowledge scales and reported their past month engagement with promotional offers. Before submitting their responses, participants also recompleted the carelessness and attention measure outlined above, with no participants failing at this stage.

### Statistical analysis

A series of mixed model analysis of variances (ANOVAs) were conducted to examine the effects of the video intervention on scepticism, persuasion knowledge and engagement with promotional offers. Ordinal data relating to past‐month engagement with promotional offers were treated as scale data for the purposes of analysis [62]. The time of testing (pre‐test, post‐test or follow‐up scores) for these measures operated as the within‐subjects variable, and the between‐subjects variables were condition (intervention and control) and PGSI category (no‐risk, low‐risk, moderate‐risk and high‐risk). Simple effects were tested as required, for example comparing change from pre‐ to post‐ in the intervention group only. Although pre‐ versus post‐test analyses were conducted on the full sample, any analyses comparing pre‐ or post‐test to follow‐up could only be conducted on the subsample who completed the follow‐up. Therefore, there are slightly different pre‐ and post‐test means reported for analyses on the full sample versus analyses on those who completed follow‐up. We report estimated marginal means (EMM) and SE because they are better suited for mixed ANOVAs. All analyses were conducted using SPSS version 28.

## RESULTS

An attrition analysis was performed on the subset of participants (*n* = 797) who were involved in the 1‐month follow‐up. The analysis revealed no significant difference in attrition between the intervention group (195/595) and the control group (208/605), with c^2^ [1] =0.35, *P* = 0.556. Furthermore, 2 × 2 between‐subjects ANOVAs revealed non‐significant interactions between condition and follow‐up status for pre‐test scepticism [*F*(1,1196) = 0.03, *P* = 0.871, η_p_^2^ = 0.00] and persuasion knowledge [*F*(1,1196) = 0.01, *P* = 0.931, η_p_^2^ = 0.00]. Therefore, attrition bias for these variables at 1‐month follow‐up appeared unlikely.

### Scepticism toward gambling advertising

Three‐way interactions showed no variation in pre‐test to post‐test changes by PGSI category within each condition, therefore, analyses were instead conducted on the conditions overall. The intervention group experienced a statistically significant increase in mean scepticism scores from pre‐test to post‐test (*P* < 0.001), whereas the control group's scores remained stable, resulting in a significant difference between groups at post‐test with a large effect size (η_p_^2^ = 0.17, *P* < 0.001). Although the intervention group's scores significantly decreased by 2.06 points at follow‐up, they remained significantly higher than the control group's (*P* < 0.001) and showed significantly greater change (*P* < 0.001, moderate effect size η_p_^2^ = 0.08). Importantly, the intervention group's follow‐up scores were still significantly higher than their pre‐test scores (*P* < 0.001), whereas the control group showed no significant changes throughout the study period (Table 2).

**TABLE 2:** Within and between subjects differences for scepticism, persuasion knowledge and promotional offer engagement for each condition across the three time‐points.

Variable	Condition	Pre‐test EMM (SE)	Post‐test EMM (SE)	1‐month follow‐up EMM (SE)	Intervention vs. control	
					Post‐test	1‐month follow‐up
Scepticism	Intervention	34.69 (0.25)	40.32 (0.25)^a^	38.26 (0.30)^b^	***P*** **< 0.001; 95% CI = [4.90–6.29]; η**_**p**_^**2**^ **= 0.17**	***P* < 0.001; 95% CI = [2.70–4.36]; η**_**p**_^**2**^ **= 0.08**
	Control	34.36 (0.26)	34.72 (0.25)	34.73 (0.30)		
Persuasion knowledge	Intervention	16.71 (0.17)	20.77 (0.16)^a^	18.63 (0.19)^b^	***P* < 0.001; 95% CI = [3.61–4.50]; η**_**p**_^**2**^ **= 0.21**	***P* < 0.001; 95% CI = [0.88–1.95]; η**_**p**_^**2**^ **= 0.03**
	Control	16.83 (0.17)	16.71 (0.16)	17.21 (0.19)		
Past‐month engagement with promotional offers	Intervention		0.73 (0.05)	0.48 (0.05)^b^	*P* < 0.244; 95% CI = [−0.23 to −0.06]; η_p_^2^ = 0.00	***P* < 0.001; 95% CI = [−0.53 to −0.26]; η**_**p**_^**2**^ **= 0.04**
	Control		0.81 (0.05)	0.87 (0.05)		

Abbreviation: EMM, estimated marginal means.

Bold represents the significant results.

^a^ Differed significantly to pre‐test within condition (*P* < 0.001).

^b^ Differed significantly to pre‐test and post‐test within condition (*P* < 0.001).

### Knowledge of persuasive strategies in gambling advertising

**FIGURE 3:**
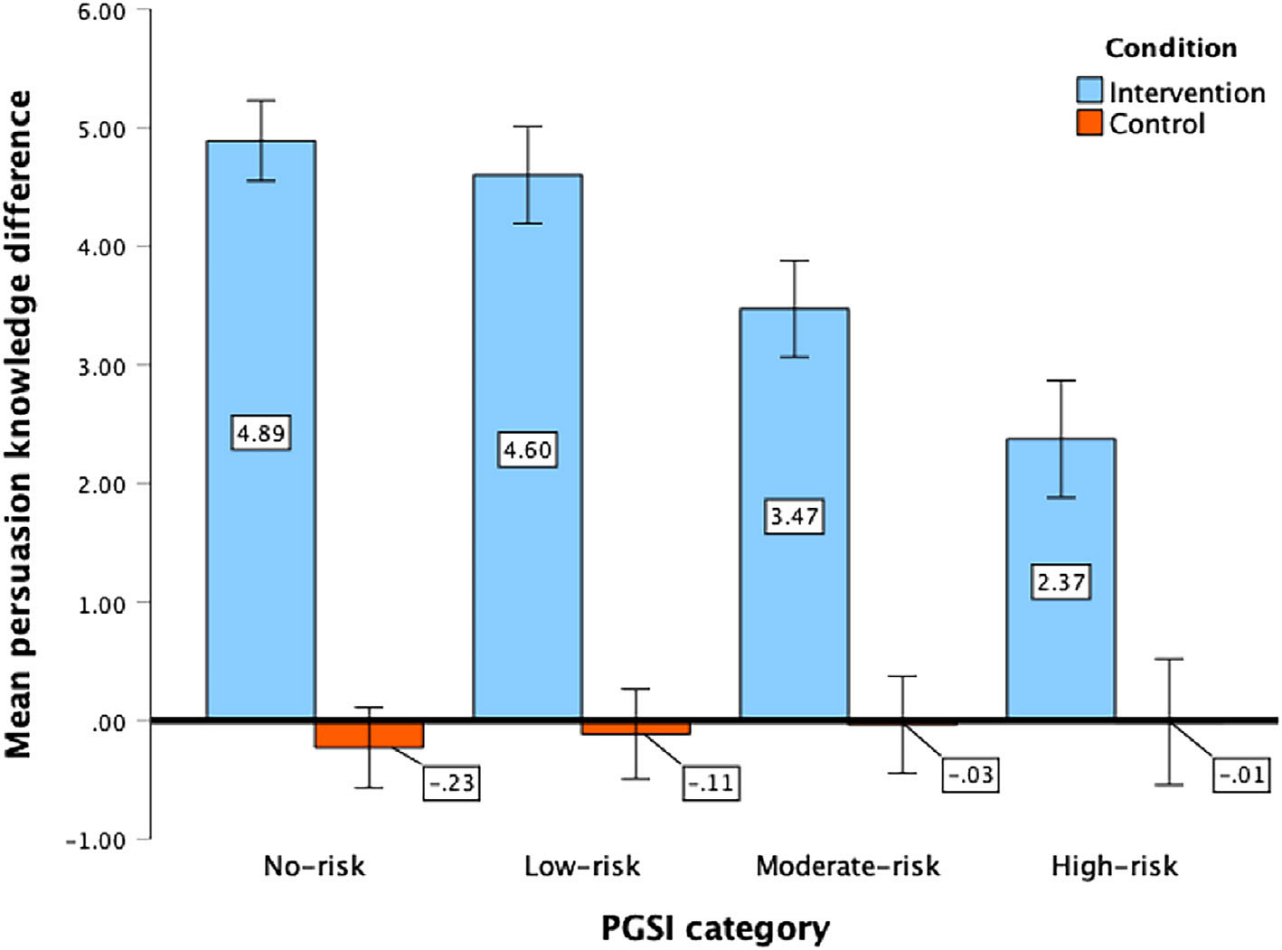
Mean difference in persuasion knowledge scores from pre‐ to post‐test by condition and PGSI category (error bars indicate 95% CI) a positive score indicates an increase in persuasion knowledge from pre‐ to post‐test. PGSI, Problem Gambling Severity Index.

Unlike the scepticism results, a significant three‐way interaction was observed by PGSI category [*F*(3, 1192) = 15.74, *P* < 0.001, η_p_^2^ = 0.04] indicating that although all PGSI categories in the intervention condition showed a significant increase in persuasion knowledge scores from pre‐ to post‐test (*P* < 0.001), the increase was smaller for higher‐risk PGSI categories (Figure 3). In relation to the conditions overall, the intervention group demonstrated a statistically significant increase in mean persuasion knowledge scores from pre‐test to post‐test (*P* < 0.001), whereas the control group's scores remained stable. Consequently, there was a significant difference between groups at post‐test with a large effect size (η_p_^2^ = 0.21, *P* < 0.001). Despite a significant decrease of 2.14 points at follow‐up, the intervention group's scores remained significantly higher than the control group's (*P* < 0.001) and showed significantly greater change (*P* < 0.001, small effect size η_p_^2^ = 0.03). Notably, the intervention group's follow‐up scores were still significantly higher than their pre‐test scores (*P* < 0.001), whereas the control group exhibited no significant changes throughout the study period (Table 2).

### Past‐month engagement with promotional offers

The intervention group demonstrated a statistically significant decrease in past‐month engagement with promotional offers from post‐test to follow‐up (*P* < 0.001). Additionally, 21% of those in the intervention group who reported engagement at post‐test completely ceased by follow‐up. In contrast, the control group's scores remained stable and showed no significant changes during this period. The difference in changes between the two groups from post‐test to follow‐up was statistically significant (*P* < 0.001), although the effect size was small (η_p_^2^ = 0.04).

## DISCUSSION

Inoculative interventions have proven effective in building resistance against the persuasive strategies of the alcohol [1, 2], tobacco [3, 4, 5] and other harmful industries [6]. The present work is the first that we know of adapting inoculation theory‐based interventions to gambling advertising. This is an important line of enquiry given the well‐established playbook of persuasive advertising strategies commonly used by the gambling industry [19, 59]. Our findings indicate that the brief intervention video successfully increased resistance against persuasive gambling advertising strategies, which was sustained after a month compared to pre‐test. In this study, resistance was operationalised via three constructs: scepticism, persuasion knowledge and disengagement with promotional offers. The intervention effects on each construct are discussed below.

The intervention video was successful in increasing scepticism toward gambling advertising strategies. Consumer scepticism is a key factor that allows individuals to resist advertising by fostering critical evaluation of advertising claims, emotional appeals and the authenticity of promoted products [55, 63, 64]. All of these facets of advertising persuasion are embodied by gambling advertising [19, 59], and our findings, therefore, underscore the importance of encouraging consumer scepticism in this context.

The intervention video was also effective in significantly increasing persuasion knowledge of gambling advertising strategies. Knowledge of persuasive strategies often operates in tandem with scepticism and allows consumers to deal with persuasive attempts made by marketers, and to develop coping mechanisms to resist them [57]. In the context of gambling advertising, consumers have expressed negative perceptions around being ‘misled’ and ‘deceived’ by persuasive attempts made by the gambling industry [18]. However, it is common for consumers to also underestimate the impact of gambling advertising persuasion on themselves, and instead perceive such advertisements as being more impactful on others [65]. The intervention video effectively addresses this discrepancy by helping consumers to recognize and assess the persuasive techniques used in gambling advertising and the effects on their own decisions more accurately.

Our significant findings are not limited to the cognitive resistance underlying scepticism and persuasion knowledge, but also extend to self‐reported behavioural effects via the decreased use of promotional offers. Despite the intervention's relative brevity, a full 21% of gamblers who reported engaging with gambling promotions at baseline had stopped doing so a month later. This suggests that longer or repeated interventions could be an effective counter to the persuasive elements within gambling advertising, and this work can inform the efforts of independent bodies promoting gambling harm prevention. For example, despite remaining higher compared to pre‐test levels and those observed in the control group, scepticism and persuasion knowledge scores declined from post‐test to follow‐up among the intervention group. This decline may be because of single exposure to the intervention video. Akin to the more continuous nature of other inoculative campaigns [1], more frequent exposure could potentially prevent this decrease in scores. We, therefore, recommend this as a future research priority to determine if repeated exposure to inoculative intervention videos further increase scepticism and persuasion knowledge over a longer period of time.

We also recommend future research that evaluates other formats of inoculative videos for gambling advertising, such as short‐form videos delivered via social media [60]. This approach might involve deconstructing inoculative videos to address individual persuasive strategies, rather than addressing multiple strategies simultaneously. This focused approach would allow for a more precise evaluation of each topic's impact and help improve future inoculative resources.

### Limitations

This study has various limitations. First, our decision to measure change in self‐reported variables via a pre‐post design may have been subject to demand effects. Including a range of distractor variables among a broader set of questionnaires may have obscured participants' awareness of our primary outcome variables. However, we aimed to reduce participant fatigue and deception by requiring participants to complete the same brief questionnaires immediately before and after video exposure. The likelihood that some participants may have inferred the study hypotheses should, therefore, be considered when interpreting our findings here. Similarly, the behavioural measure of past‐month engagement with promotional offers was reliant on the recall of participants, which may also be prone to demand effects and self‐report bias [66]. Controlled laboratory experiments on gambling advertising impact are notoriously difficult to conduct because of methodological issues and ethical concerns [67]. Future research could more accurately assess engagement with promotional offers via the gold‐standard approach of analysing gamblers' account data [66, 68]. Second, and relatedly, in anticipation of participant dropout, we did not conduct an intention‐to‐treat (ITT) analysis. This is a limitation as only a subset (*n* = 797) of participants were included in the 1‐month follow‐up. The analysis of account data would, therefore, also mitigate this issue in future research. Third, this study provided follow‐up measures after 1‐month only, and any observed effects may weaken over longer timescales. Fourth, the design of this experiment involved financial motivation for participants to view either the intervention or control video. Consequently, we cannot be certain that viewers would choose to watch the full 7‐minute intervention video of their own volition if they came across it naturally on the internet. Fifth, this experiment used a crowd‐sourced sample of young adult gamblers via the platform Prolific. This sample may be prone to self‐selection bias [69], and may not be representative of other age groups, nor the wider population. However, these biases are hard to avoid in gambling research and crowd‐sourcing methods allow access to larger proportions of people who are in higher‐risk groups [70]. Sixth, this study did not compare the intervention video against other independently developed campaigns, which might have been effective [71].

## CONCLUSIONS

An inoculative video intervention promoted scepticism and persuasion knowledge of gambling advertising strategies among young adult gamblers and led to a self‐reported decrease in engagement with gambling promotions over the following month. Inoculative gambling interventions should be used and further improved on within efforts to prevent gambling‐related harm.

## AUTHOR CONTRIBUTIONS

**Jamie Torrance:** Conceptualization (equal); data curation (lead); formal analysis (equal); funding acquisition (equal); investigation (lead); methodology (lead); project administration (lead); writing—original draft (lead). **Alex M. T. Russell:** Data curation (equal); formal analysis (equal); investigation (equal); methodology (equal); validation (equal); writing—original draft (equal). **Conor Heath:** Investigation (equal); methodology (equal). **Philip Newall:** Conceptualization (equal); data curation (equal); formal analysis (equal); funding acquisition (equal); investigation (equal); methodology (equal); project administration (equal); supervision (lead); writing—original draft (equal).

## DECLARATIONS OF INTEREST

The authors declare that there are no competing interests in relation to this manuscript. In the last 3 years, J.T. has received; (1) PhD funding from GambleAware; (2) open access publication funding from Gambling Research Exchange Ontario (GREO); (3) paid consultancy fees from Channel 4; (4) conference travel and accommodation funding from the Academic Forum for the Study of Gambling (AFSG); and (5) A minor exploratory research grant from the AFSG and GREO. In the last 3 years, A.M.T.R. has received funding from the Victorian Responsible Gambling Foundation; the New South Wales Office of Responsible Gambling; the South Australian Government; the Australian Capital Territory Government; Gambling Research Australia; and the New Zealand Ministry of Health. He has had travel expenses paid to present research by the Victorian Responsible Gambling Foundation, PsychMed and the Hawthorn Hawks Football Club Players Association. He has received an honorarium from Movember for assessing applications for funding and consulting fees from the Victorian Responsible Gambling Foundation. C.H. has no disclosures to make. P.N. is a member of the Advisory Board for Safer Gambling—an advisory group of the Gambling Commission in Great Britain. In the last 3 years, P.N. has contributed to research projects funded by the Academic Forum for the Study of Gambling, Clean Up Gambling, Gambling Research Australia, New South Wales Responsible Gambling Fund and the Victorian Responsible Gambling Foundation. P.N. has received travel and accommodation funding from Alberta Gambling Research Institute and the Economic and Social Research Institute and received open access fee funding from GREO.

## Data Availability

The preregistration can be found here: (https://osf.io/gk3by), with the data, materials, and intervention video available from https://osf.io/jb43f/.

## Footnotes

1 The PGSI categories associated with the lowest and highest gambling‐risk have previously been represented via the labels ‘non‐problem’ and ‘problem gambler’ respectively. However, to avoid stigmatising language, they will here be referred to as ‘no‐risk’ and ‘high‐risk’.
